# Relationship between somatic cell counts and subclinical mastitis in lactating dairy cows

**DOI:** 10.14202/vetworld.2020.1709-1713

**Published:** 2020-08-27

**Authors:** S. M. Mostafizur Rahaman Sumon, Mst. Sonia Parvin, Md. Amimul Ehsan, Md. Taohidul Islam

**Affiliations:** 1 Population Medicine and AMR Laboratory, Department of Medicine, Faculty of Veterinary Science, Bangladesh Agricultural University, Mymensingh 2202, Bangladesh; 2 Department of Medicine, Faculty of Veterinary Medicine and Animal Science, Bangabandhu Sheikh Mujibur Rahman Agricultural University, Gazipur 1706, Bangladesh

**Keywords:** Bangladesh, bovine mastitis, intramammary infection, somatic cell count threshold, sensitivity, specificity

## Abstract

**Background and Aim:**

Subclinical mastitis (SCM) is one of the most prevalent diseases of dairy cows, and somatic cell count (SCC) is widely used to determine SCM and milk quality. However, setting the threshold of SCC is very important. This study aimed to determine the cow-level threshold of SCC to differentiate milk of SCM affected cows from normal milk in Bangladesh.

**Materials and Methods:**

Sensitivity (Se) and specificity (Sp) along with other characteristics of different thresholds of SCC were determined considering the bacterial culture as the gold standard test. Three definitions of intramammary infection (IMI) were set based on the group of pathogens involved. Five categories of SCC thresholds were considered for analysis.

**Results:**

Se and Sp of thresholds of SCC greatly varied as definitions of IMI changed. Irrespective of SCC thresholds, Se increased when isolation of major pathogens included in IMI definitions. Se decreased when SCC thresholds increased (from 100 to 300 × 10^3^ cells/mL) for all IMI definitions and ranged from 60.6% to 20.3%. The highest Se was found at low SCC threshold (100 × 10^3^ cells/mL), which resulted in less false-negative outcome. On the other hand, Sp increased with the increment of SCC thresholds giving rise to a less false-positive outcome.

**Conclusion:**

The cow-level SCC threshold of 100 × 10^3^ cells/mL was found appropriate to identify subclinical IMI of dairy cows.

## Introduction

Mastitis is the most prevalent and important disease of dairy cows and responsible for the major economic losses to the dairy farms globally [1]. It is a multicausal disease, and various pathogens are involved in subclinical and clinical forms of mastitis [2,3]. Subclinical mastitis (SCM) is characterized by high somatic cell count (SCC) in milk without any visible abnormalities of the milk and udder and obvious systemic signs [3]. The occurrence of SCM is very common in dairy herds compared to clinical mastitis, which results in huge economic losses. It was estimated that decreased milk yield due to SCM caused losses of about 122.6 (US$ 2.11) million Taka annually [4]. In addition, SCM affected cows remain as the source of infection to other cows in the herd.

SCM is most frequently the results of bacterial intramammary infection (IMI), therefore, the terms IMI and SCM are often used interchangeably [5]. The IMI is defined by the presence of any pathogens in the mammary quarter [6]. In general, IMI is predicted by the elevation of SCC, and also, the milk quality and mastitis control program are assessed based on SCC [7]. Somatic cells are mainly comprised of udder epithelial cells and white blood cells, that are, neutrophils, phagocytes, and lymphocytes, and can indicate IMI when elevated [8]. Consequently, it is essential to establish SCC threshold, which is a value that theoretically differentiates uninfected cows from the infected ones, and high-quality information is required on the operating characteristics (e.g., *Se* and *Sp*) to interpret the test results. SCM is often diagnosed on the basis of SCC values that exceed the threshold level. Several SCC cutoffs have been evaluated to distinguish between infected and non-infected mammary glands and to make management decisions. A mammary quarter is deemed to be healthy when the SCC level is usually lower than 100,000 cells/mL of milk [9,10]. Conceivably under field conditions, to reduce the diagnostic error, SCC below 200,000 cells/mL of milk is widely used as the threshold level to differentiate healthy udders from subclinically affected ones [11]. Some reports suggest that milk with high SCCs may not confirm the invasion of mammary quarter with mastitis pathogens in some cases, and conversely, it is also conceived that there is the presence of pathogens in milk with very low SCCs [11-13]. However, SCC is still of great value as a screening test to diagnose SCM provided the results are interpreted very carefully considering the demographic characteristics of cows.

Although the provision of SCC is readily available to most dairy farms in developed countries on a monthly basis through the dairy herd improvement (DHI) program, this practice yet not accustomed in Bangladesh. Because of the high prevalence (30-72%) of SCM and abundance of the mastitis pathogens [14-19], the provision of SCCs for routine SCM screening in dairy herds will be of great benefit in reducing the occurrence of SCM and ensuring milk quality as well.

However, no precise and comprehensive reports are available on the *Se* and *Sp* of SCC thresholds to determine SCM of dairy cows in Bangladesh. Considering this fact, this study aimed to determine the cow-level threshold of SCC for the diagnosis of SCM affected cows.

## Materials and Methods

### Ethical approval and Informed consent

There was no need for ethical approval as the study was carried out on milk of cows reared in different groups on the farm and generally fed with different locally available grasses and concentrates in different seasons. Moreover, the authors declare that they have obtained written informed consent from the owner of the animals to collect milk samples from cows along with data.

### Study period, selection of cows, collection and transportation of samples

Ninety healthy cross-bred cows on lactation from Bangladesh Agricultural University Dairy Farm and smallholder dairy farms of Mymensingh Sadar Upazila were randomly selected, and subsequently, 360 mammary quarters were sampled aseptically for the collection of milk in Falcon tubes during the period from July 2016 to March 2017. The age, number of lactation, and lactation stage of cows were 2.5-8 years, 1-7, and 10-350 days, respectively. Before collection, cleaning followed by disinfection of the teat was done using 70% ethanol soaked cotton. Approximately 10 mL of milk sample was collected from each mammary quarter after discarding the first two to three streams of milk. Cows with visible abnormalities in the udder and milk, systemic signs of mastitis, and the history of any antimicrobial treatment during the past 7 days onward were excluded from the study. Moreover, cows at the 1^st^ week of lactation were not included because SCCs normally remain high in milk during this period.

### SCCs

Somatic cells in milk samples were counted using NucleoCounter^®^ SCC-100^™^ (ChemoMetec) as per the manufacturer’s instruction. Briefly, equal volume of milk and reagent (50 µL each) were pre-diluted and then loaded in cassette and reading was taken.

### Isolation and identification of bacteria

All the samples were examined bacteriologically as described earlier [20]. Briefly, within 24 h after collection, about 10 μL of milk was inoculated onto 5% sheep blood agar plate in triplicate, then incubated aerobically for 48 h. Positive culture was subcultured onto different selective media such as mannitol salt agar, MacConkey agar, EMB agar, cetrimide agar, and triple sugar iron slants. Bacteria were identified by means of colony morphology, Gram staining properties, hemolytic properties on blood agar, and properties of different biochemical reactions (e.g., catalase, coagulase, oxidase, methyl red/Voges–Proskauer, indole, and sugar fermentation) as described earlier [21]. Bacteria were grouped into three categories, (1) major pathogens that included *Staphylococcus aureus*, *Escherichia coli*, and *Enterobacter* spp., (2) minor pathogens, which consisted of coagulase-negative *Staphylococcus*, and (3) other pathogens that comprised *Bacillus* spp., *Pseudomonas aeruginosa*, and unidentified bacteria as reported earlier [19].

### IMI

Three definitions of IMI were set based on the group of pathogens involved. Definition I: If there was a growth of any of the major pathogens, the quarter was considered infected; Definition II: If there was a growth of any of the major and/or minor pathogens; Definition III: If there was a growth of any major and/or minor and/or other pathogens [22].

### Statistical analysis

The validity of five various thresholds level of SCC (100, 150, 200, 250, and 300 × 10^3^ cells/mL) was evaluated against bacterial culture for diagnosing IMI using the following quality parameters.

Se = TP/(TP+FN)
Sp = TN/(TN+FP)
PPV =TP/(TP+FP)
NPV = TN/(TN+FN)
Where,
TP = true-positive test result (infected quarter above SCC threshold)
FP = false-positive test result (uninfected quarter above SCC threshold)
TN = true-negative test result (uninfected quarter below or equal to SCC threshold)
FN = false-negative test result (infected quarter below or equal to SCC threshold)

Sensitivity (*Se*) was the proportion of infected quarters that had SCC values above the selected threshold, and specificity (*Sp*) was the proportion of uninfected quarters that had SCC values below the selected threshold. Positive predictive value (PPV) was the proportion of truly infected quarters to the test positive quarters and negative predictive value (NPV) was the proportion of truly uninfected quarters to the test negative quarters. Descriptive statistics such as frequency and confidence intervals were performed using the statistical software SPSS v.22.

## Results

The bacteriological analysis revealed that 35.6% of samples were culture positive; that is, the quarter-level prevalence of IMI was 35.6%. However, no growth was observed in 64.4% of samples. Major pathogens were isolated from 32.8% of samples while minor pathogens from 17.2% of samples. *Se*, *Sp*, and predictive values at different levels of SCC thresholds are presented in Table-1.

**Table-1:** Sensitivity, specificity, and predictive values (positive and negative) of five SCC thresholds (100-300×10^3^cells/mL) to identify IMI, considering three criteria of IMI of dairy cows.

Criteria and SCC threshold	Sensitivity, % (95% CI)	Specificity, % (95% CI)	Predictive value^1^, % (95% CI)	

			PPV	NPV
IMI = only major pathogens; UMQ^2^= only culture-negative samples				
100×10^3^	60.6 (54.8, 66.4)	95.7 (95.2, 96.2)	80.0 (75.6, 84.4)	89.5 (88.3, 90.7)
150×10^3^	48.5 (42.5, 54.5)	96.6 (96.2, 97.0)	80.0 (75.0, 84.9)	86.8 (85.4, 88.2)
200×10^3^	36.4 (30.8, 41.9)	96.6 (96.2, 97.0)	75.0 (68.5, 81.5)	84.2 (82.6, 85.8)
250×10^3^	27.3 (22.5, 32.1)	97.4 (97.1, 97.7)	75.0 (67.5, 82.5)	82.5 (80.8, 84.2)
300×10^3^	21.2 (17.2, 25.2)	97.4 (97.1, 97.7)	70.0 (60.8, 79.2)	81.3 (79.5, 83.1)
IMI = only major pathogens; UMQ = minor pathogen, others and culture-negative samples				
100×10^3^	60.6 (54.8, 66.4)	87.1 (85.8, 88.4)	51.3 (45.8, 56.8)	90.8 (89.8, 91.8)
150×10^3^	48.5 (42.5, 54.5)	91.8 (90.9, 92.7)	57.1 (50.7, 63.5)	88.8 (87.7, 89.9)
200×10^3^	36.4 (30.8, 41.9)	92.5 (91.7, 93.3)	52.2 (44.9, 59.4)	86.6 (85.3, 87.8)
250×10^3^	27.3 (22.5, 32.1)	93.2 (92.5, 93.9)	47.4 (39.5, 55.3)	85.1 (83.7, 86.5)
300×10^3^	21.2 (17.2, 25.2)	93.9 (93.2, 94.6)	43.8 (35.3, 52.3)	84.1 (82.7, 85.5)
IMI = major and minor pathogens; UMQ = only culture-negative samples				
100×10^3^	56.8 (51.7, 61.9)	95.7 (95.2, 96.2)	83.3 (79.8, 86.8)	85.4 (83.9, 86.9)
150×10^3^	40.9 (35.8, 45.9)	96.6 (96.2, 97.0)	81.8 (77.4, 86.2)	81.2 (79.4, 83.0)
200×10^3^	31.8 (27.3, 36.3)	96.6 (96.2, 97.0)	77.8 (72.2, 83.4)	78.9 (76.9, 80.8)
250×10^3^	25.0 (21.1, 28.9)	97.4 (97.1, 97.7)	78.6 (72.4, 84.8)	77.4 (75.4, 79.4)
300×10^3^	20.5 (17.1, 23.9)	97.4 (97.1, 97.7)	75.0 (67.5, 82.5)	76.4 (74.3, 78.5)
IMI = major, minor, and other pathogens; UMQ= only culture-negative samples				
100×10^3^	53.1 (48.8, 57.4)	95.7 (95.2, 96.2)	87.2 (84.7, 89.7)	78.7 (76.7, 80.7)
150×10^3^	37.5 (33.4, 41.6)	96.6 (96.2, 97.0)	85.7 (82.5, 88.9)	73.7 (71.5, 75.9)
200×10^3^	29.7 (26.1, 33.3)	96.6 (96.2, 97.0)	82.6 (78.4, 86.8)	71.3 (69.0, 73.6)
250×10^3^	25.0 (21.8, 28.2)	97.4 (97.1, 97.7)	84.2 (79.9, 88.4)	70.2 (67.9, 72.5)
300×10^3^	20.3 (17.5, 23.1)	97.4 (97.1, 97.7)	81.3 (75.9, 86.5)	68.9 (66.6, 71.2)

1 PPV=Positive predictive value, NPV=Negative predictive value,

2 UMQ=Uninfected mammary quarters, CI=Confidence interval

*Se* was decreased (60.6-20.3%) with the increase of SCC thresholds for all IMI definitions. However, there was an increase in *Se* for all SCC threshold levels when IMI definition was restricted only to the presence of major pathogens. For an SCC threshold of 100 × 10^3^ cells/mL of milk, *Se* was 60.6% versus 53.1% (IMI Definition I vs. III), and for threshold of 200 × 10^3^ cells/mL, *Se* was 36.4% versus 29.7% (IMI Definition I vs. III).

In this study, *Sp* ranged from 87.1 to 97.4% for different SCC thresholds and IMI definitions. There was an increase in *Sp* when culture-negative results were interpreted as the absence of IMI at all SCC threshold levels. At SCC thresholds of 100 × 10^3^ cells/mL and 200 × 10^3^ cells/mL, *Sp* values were 95.7% and 96.6%, respectively, when uninfected mammary quarters were defined on the basis of culture-negative results only. However, the *Se* was high at low SCC threshold (i.e., 100 × 10^3^ cells/mL), which resulted in less false-negative outcome. On the contrary, *Sp* was increased with the increment of SCC thresholds, which resulted in least false-positive outcome. PPVs and NPV ranged from 43.8% to 87.2% and 68.9-90.8%, respectively, depending on SCC thresholds and IMI definitions.

## Discussion

This is the first report on SCC threshold at cow-level for the diagnosis of SCM of dairy cows in Bangladesh where mastitis pathogens are abundant. As SCM is most often due to bacterial IMI [5], we calculated the SCC thresholds taking the IMI as the gold standard test. The results can be extrapolated in other developing countries where dairy farming system and abundance of mastitis pathogens are as alike as Bangladesh. As errors may arise in any diagnostic test, particularly when reliance is placed on a single screening profile [23], we calculated *Se* and *Sp* at five levels of SCC thresholds here in this study.

The results of this study indicated that *Se* was decreased with the increment of SCC thresholds for all IMI definitions. However, *Se* values increased at all SCC thresholds when IMI definition was restricted to only major pathogens. It might be due to the inclusion of all types of pathogens (minor and other pathogens in addition to major pathogens) in IMI definition that might have resulted in false-negative outcome as minor and other pathogens seldom cause severe mammary inflammation other major pathogens [12]. This result is in agreement with the earlier report in which *Se* at SCC threshold of 100 × 10^3^ cells/mL was the highest (74.0%) when only major pathogens were associated with IMI and lowest (66.4%) when both the major and minor pathogens were taken into account as the causes of IMI [24].

*Sp* increased with the increase of SCC thresholds for all IMI definitions. This results well correspond with the earlier observation in which *Sp* values were increased from 50.8 to 81.2% with the increase of SCC thresholds and for different IMI definitions [24]. Both *Se* and *Sp* were considerably higher for distinguishing IMI with major pathogens from other pathogens, which is compatible with previous reports [25].

Both PPV and NPV ranged from 43.8 to 87.2% and 68.9 to 90.8%, respectively, depending on SCC thresholds and IMI definitions. The findings of high PPV may possibly be due to the high prevalence of IMI with major pathogens. Besides, the culture results of either pooled, single, duplicate, or triplicate milk samples to define IMI may also influence the predictive values as described earlier [22,26].

High *Se* at low SCC thresholds and increased *Sp* with the increase of SCC thresholds found in our study are consistent with earlier observations [27]. We found maximum *Se* and minimum *Sp* at SCC threshold of 100 × 10^3^ cells/mL and for all IMI definitions. It is likely that use of SCC threshold value of 200 × 10^3^ cells/mL instead of threshold of 100 × 10^3^ cells/mL might account higher occurrence of false-negative outcome, that is, some truly infected cows with low SCC could be identified as non-infected. The occurrence of such false-negative results may adversely affect the implementation of a mastitis control program in farms because some cows could not be correctly identified as a source of infection. Thus, based on the findings, it was evident that the SCC threshold level of 100 × 10^3^ cells/mL could be considered to detect the subclinically infected mammary quarters and to lessen the possibility of false-negative outcome. The results are consistent with the earlier recommendation [24]. Other researchers also proposed the threshold level of 100 × 10^3^ cells/mL [22,25].

Although in detecting IMI, culture of bacteria is deemed a reliable process, isolation of pathogens may be affected by various factors such as type of bacteria, amounts of bacteria in milk along with intermittent excretion nature, frequency of sampling, storage condition of sample, volume of milk plated, and presence of some enzymes/proteins [22,28]. In this context, bacterial culture in triplicate could be ideal for detecting IMI but not cost-effective [29]. In an attempt to minimize the cost, culture of single milk sample could also be worthwhile for the detection of IMI. Reports also suggest that the culture of a single milk sample is allowable to determine IMI caused by most of the mastitic pathogens since the *Se* and *Sp* were quite higher (85.8% and 75.1%) in comparison to the culture results of milk in triplicate [22]. Therefore, the cow-level SCC threshold determined in our study based on single milk sampling would be applicable for all tropical countries as like as Bangladesh.

## Conclusion

*Se* is increased when the threshold level of SCC is lowered, and *Sp* increased when the threshold is raised. However, increased *Se* results in less false-negative outcome while increased *Sp* gives minimal false-positive outcome. Based on the findings, the cow-level SCC threshold of 100 × 10^3^ cells/mL that results in less false-negative outcome could be appropriate to identify the IMI of lactating dairy cows in countries like Bangladesh where mastitis pathogens are abundant.

## Authors’ Contributions

SMMRS was involved in designing and conducting the study, analyzing data, and drafting the manuscript. MSP contributed to conducting the study, analyzing data, and revising the manuscript. MAE contributed to the study design and revision of the manuscript. MTI was involved in conceptualizing, supervising, designing, and coordinating the study, interpreting data, and revising the manuscript.

## Competing Interests

The authors declare that they have no competing interests.

## Publisher’s Note

Veterinary World remains neutral with regard to jurisdictional claims in published institutional affiliation.
